# Supplementary material for: Identification and functional analysis of non-coding regulatory small RNA FenSr3 in Bacillus amyloliquefaciens LPB-18
Source: PeerJ. 2023 May 15;11:e15236. doi: 10.7717/peerj.15236 (PMC10194069; doi:10.7717/peerj.15236)
Supplement: Supplemental Information 4 [file peerj-11-15236-s004.zip › KO/CK-vs-T1_map/map00965.html]

KEGG PATHWAY: Betalain biosynthesis - Reference pathway


|  |  |
| --- | --- |
| **Betalain biosynthesis - Reference pathway** |  |

[
Pathway menu
| Organism menu
| Pathway entry
| Show description
| User data mapping
]

Betalains are water-soluble nitrogen-containing pigments that are present in plants belonging to the order Caryophyllales (such as cactus and amaranth families) and in higher fungi. They contain betalamic acid as the chromophore and are classified into two types: betacyanins and betaxanthins. Betacyanins contain a cyclo-DOPA residue and exhibit red/violet coloration, while betaxanthins contain different amino acids or amino side chains and exhibit a yellow/orange coloration. The condensation of betalamic acid with amino acids (including cyclo-DOPA or amines) in plants is a spontaneous reaction, not an enzyme-catalyzed reaction.

|  |  |  |
| --- | --- | --- |
| Reference pathway | 184% 150% 122% 100% 82% 67% 55% | 图片下载 |
